# Double-endoscope endoscopic submucosal dissection with snare traction and loop stabilization for adenoma involving appendiceal orifice

**DOI:** 10.1055/a-2197-8828

**Published:** 2023-11-21

**Authors:** Hsin-Yu Chen, Chung-Ying Lee, Chao-Wen Hsu, Jen-Hao Yeh, Tsung-Hsien Chen, Kun-Feng Tsai, Chu-Kuang Chou

**Affiliations:** 1 34903 School of Medicine, College of Medicine, Fu Jen Catholic University, Taipei, Taiwan; 2 60616 Division of Gastroenterology and Hepatology, Cathay General Hospital, Taipei, Taiwan; 3 Division of Gastroenterology and Hepatology, Department of Internal Medicine, Shuang Ho Hospital, Taipei Medical University, New Taipei City, Taiwan; 4 38032 Division of Gastroenterology and Hepatology, Department of Internal Medicine, School of Medicine, College of Medicine, Taipei Medical University, Taipei, Taiwan; 5 38032 TMU Research Center for Digestive Medicine, Taipei Medical University, Taipei, Taiwan; 6 Division of Colorectal Surgery, Kaohsiung Veterans General Hospital, Kaohsiung, Taiwan; 7 School of Medicine, National Yang-Ming University, Taipei, Taiwan; 8 Division of Gastroenterology and Hepatology, Department of Internal Medicine, E-DA DaChang Hospital, I-Shou University, Kaohsiung, Taiwan; 9 36597 Department of Internal Medicine, Ditmanson Medical Foundation Chia-Yi Christian Hospital, Chiayi City, Taiwan; 10 Department of Internal Medicine, Gastroenterology and Hepatology Section, An Nan Hospital, China Medical University, Tainan, Taiwan; 11 49048 Department of Medical Sciences Industry, Chang Jung Christian University, Tainan, Taiwan; 12 36597 Division of Gastroenterology and Hepatology, Department of Internal Medicine, Ditmanson Medical Foundation Chia-Yi Christian Hospital, Chiayi, Taiwan; 13 36597 Obesity center, Ditmanson Medical Foundation Chia-Yi Christian Hospital, Chiayi, Taiwan

Traction during colorectal endoscopic submucosal dissection (ESD) is essential for tackling difficult lesions. Although conventional clip–band-based methods are easy to use, they are difficult to adjust, require flaps, and offer limited traction forces. Using snare traction during double-endoscope ESD (DE-ESD) has proven to be effective in reducing procedure time and overcoming complex anatomical challenges 1 2 .

The 3cm 0-Is adenoma was found at the appendiceal orifice in a patient requiring peritoneal dialysis. It was challenging to trim into the submucosa and create a mucosal flap for clip-based traction because bowel folds covered the surrounding area of the lesion and colonic looping obstructed the approach axis ( Fig. 1 , Video 1 ). We inserted two endoscopes, one GIF H290 followed by GIF Q260J (Olympus, Tokyo, Japan), using the previously mentioned method 1 . The looping became more stable and maneuverability improved. Snare traction was created from the traction endoscope by snaring the lesion ( Fig. 2 ). The traction could be adjusted in real time as required ( Fig. 3 , Fig. 4 ). The procedure time was 30 minutes without any complications ( Fig. 5 ). The patient was discharged the day after ESD, and the final pathology revealed a completely resected adenoma.

**Fig. 1:**
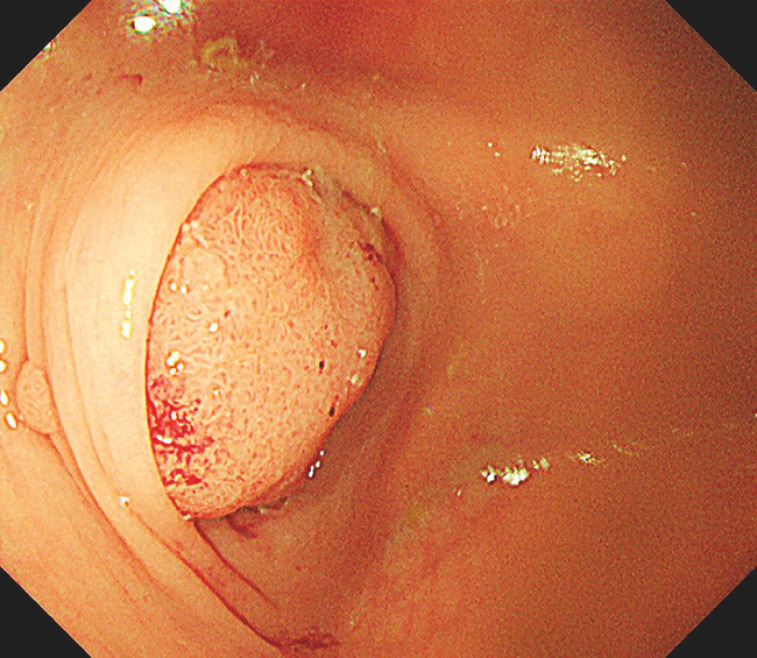
Visualization of the lesion at the appendiceal orifice.

**Fig. 2:**
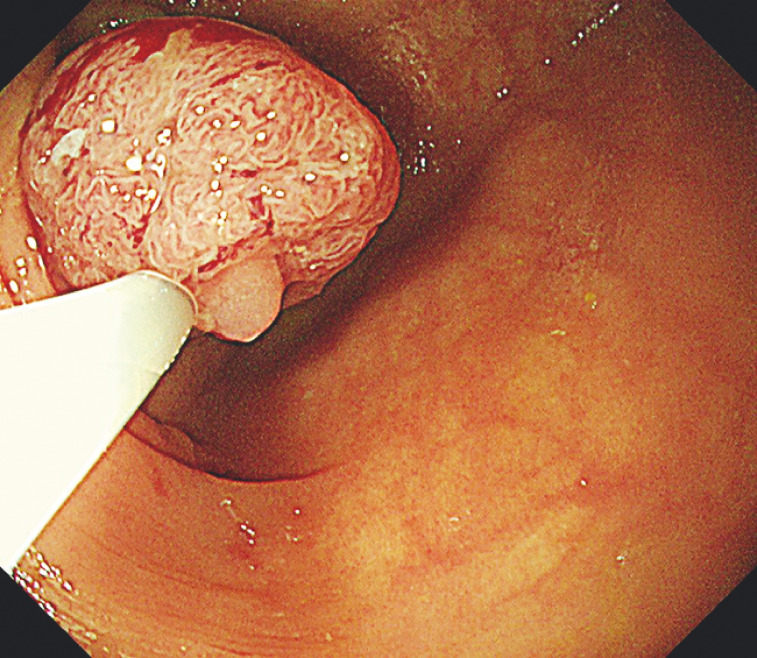
Traction endoscope view showing snaring of the lesion for enhanced traction.

**Fig. 3:**
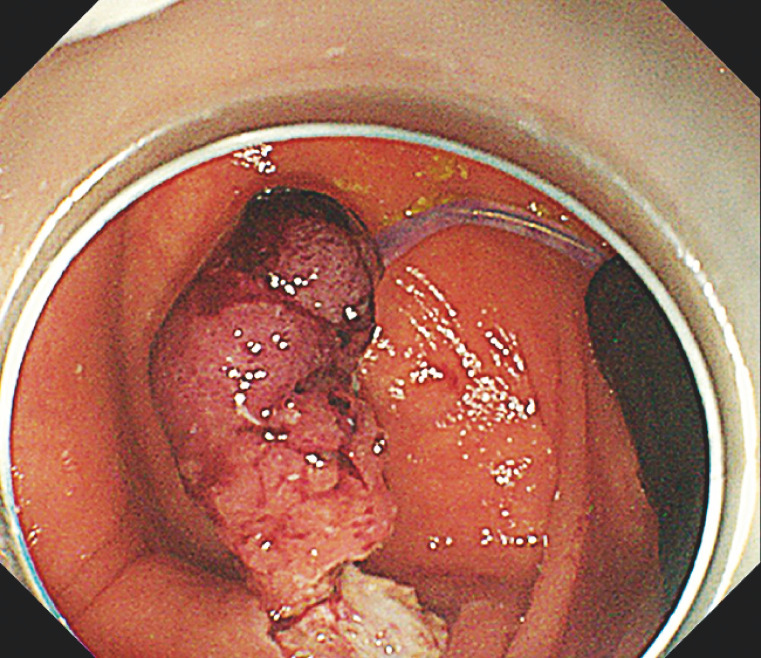
Endoscopic submucosal dissection endoscope view revealing the traction endoscope and traction snare.

**Fig. 4:**
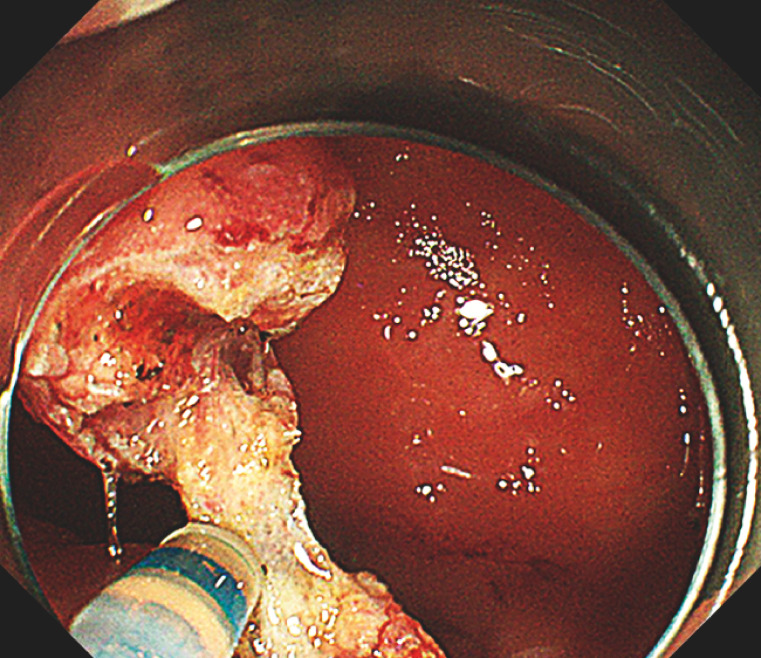
Successful extraction of the lesion from the appendiceal orifice after adjusting the traction force.

**Fig. 5:**
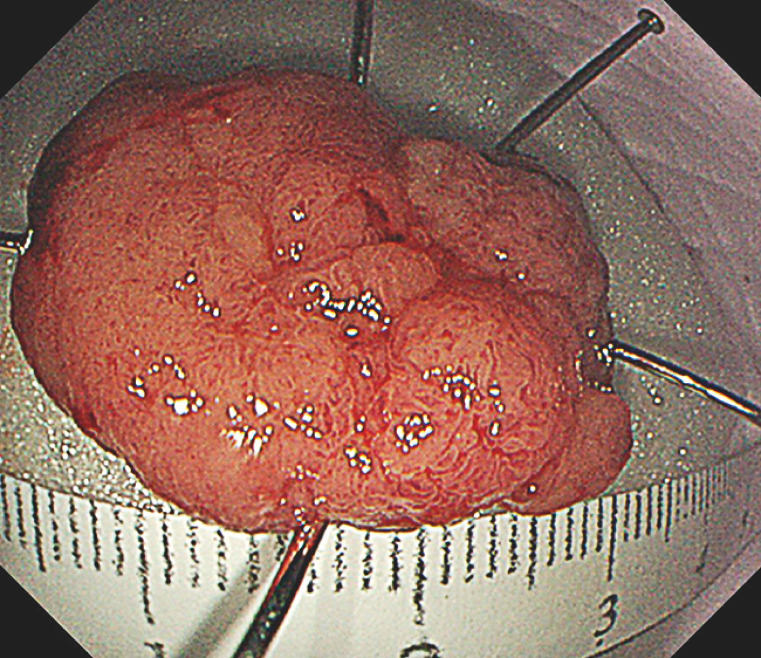
Smooth and complication-free resection of the lesion was completed within a 30-minute timeframe.

Double-endoscope endoscopic submucosal dissection with snare traction and loop stabilization for adenoma involving the appendiceal orifice.Video 1

DE-ESD can offer strong adjustable traction and stabilize the colon loop to facilitate resection. With the help of the additional endoscope, the snare traction can provide alternative options for traction that do not rely on clips.

Endoscopy_UCTN_Code_TTT_1AQ_2AD

## Notes

Endoscopy E-Videos https://eref.thieme.de/e-videos
*E-Videos* is an open access online section of the journal *Endoscopy* , reporting on interesting cases and new techniques in gastroenterological endoscopy. All papers include a high-quality video and are published with a Creative Commons CC-BY license. Endoscopy E-Videos qualify for HINARI discounts and waivers and eligibility is automatically checked during the submission process. We grant 100% waivers to articles whose corresponding authors are based in Group A countries and 50% waivers to those who are based in Group B countries as classified by Research4Life (see: https://www.research4life.org/access/eligibility/ ). This section has its own submission website at https://mc.manuscriptcentral.com/e-videos .
